# Defining Hyperphagia for Improved Diagnosis and Management of MC4R Pathway–Associated Disease: A Roundtable Summary

**DOI:** 10.1007/s13679-024-00601-z

**Published:** 2025-01-25

**Authors:** Steven B. Heymsfield, Karine Clément, Beatrice Dubern, Anthony P. Goldstone, Andrea M. Haqq, Peter Kühnen, Jesse Richards, Christian L. Roth, Erica L. T. van den Akker, Martin Wabitsch, Jack A. Yanovski

**Affiliations:** 1 Pennington Biomedical Research Center, Louisiana State University System, Baton Rouge, LA USA; 2 Nutrition Department, Assistance Publique-Hôpitaux de Paris, Pitié-Salpêtrière Hospital, Paris, France; 3 Sorbonne Université, Inserm, Nutrition and Obesities, Systemic Approaches, NutriOmique Research Group, Paris, France; 4 Sorbonne Université, Trousseau Hôpital, Assistance Publique-Hôpitaux de Paris, Paris, France; 5 PsychoNeuroEndocrinology Research Group, Division of Psychiatry, Department of Brain Sciences, Faculty of Medicine, Imperial College London, London, UK; 6 Imperial Centre for Endocrinology, Imperial College Healthcare NHS Trust Hammersmith Hospital, London, UK; 7 Department of Pediatrics, Division of Pediatric Endocrinology, University of Alberta, Edmonton, AB Canada; 8 Department of Pediatric Endocrinology and Diabetology, Charité - Universitätsmedizin Berlin, Corporate Member of Freie Universität Berlin und Humboldt-Universität zu Berlin, Berlin, Germany; 9 German Center for Child and Adolescent Health (DZKJ), Partner Site, Berlin, Germany; 10 Department of Internal Medicine, University of Oklahoma at Tulsa, Tulsa, OK USA; 11 Seattle Children’s Research Institute, Seattle, WA USA; 12 Division of Endocrinology, Department of Pediatrics, University of Washington, Seattle, WA USA; 13 Erasmus University Medical Center, Rotterdam, The Netherlands; 14 Division of Pediatric Endocrinology and Diabetes, Department of Pediatrics and Adolescent Medicine, University of Ulm, Ulm, Germany; 15 Present Address: German Center for Child and Adolescent Health (DZKJ), Partner Site, Ulm, Germany; 16 Section on Growth and Obesity, Division of Intramural Research, Eunice Kennedy Shriver National Institute of Child Health and Human Development, National Institutes of Health, Bethesda, MD USA; 17 Pennington Biomedical Research Center, Louisianna State University, 6400 Perkins Rd, Baton Rouge, LA 70808 USA

**Keywords:** Hyperphagia, MC4R, Melanocortin-4 receptor, Obesity, Hunger

## Abstract

**Purpose of review:**

Hyperphagia is a condition associated with rare obesity-related diseases, presenting as a pathologic, insatiable hunger accompanied by abnormal food-seeking behaviors. In October 2023, a group of researchers and clinicians with expert knowledge on hyperphagia convened at the annual ObesityWeek meeting to discuss the need for a unified definition of hyperphagia and key items necessary to improve the identification, assessment, and treatment of hyperphagia in patients with melanocortin 4 receptor (MC4R) pathway–associated diseases.

**Recent findings:**

The definition of hyperphagia proposed by this group is a pathologic, insatiable hunger accompanied by abnormal food-seeking behaviors. Suggested methods to accurately identify patients with hyperphagia include increased physician and parent/caregiver education and standardized efficient screening procedures for use in the clinic. The etiology of hyperphagia as related to abnormal MC4R signaling was also reviewed and proposed as a central cause of the condition across several underlying diseases.

**Summary:**

Given this potential unified underlying pathology, the expert group recommends that patients with hyperphagia undergo genetic testing and that treatment include comprehensive weight-management strategies incorporating lifestyle and pharmacotherapies targeted at addressing hyperphagia.

## Introduction

Hyperphagia is a clinical symptom associated with some rare diseases of obesity described as an insatiable form of hunger characterized by a severe preoccupation with food [1–5]. Investigations into rare forms of obesity such as Bardet-Biedl syndrome (BBS); leptin, leptin receptor (LEPR), or proopiomelanocortin (POMC) receptor deficiencies; and acquired hypothalamic obesity have advanced our understanding of the biology of hyperphagia and its distinction from other overeating behaviors [2, 4, 6–11]. Underlying these rare forms of obesity are key signaling pathways, such as the leptin–melanocortin-4 receptor (MC4R) pathway, which centrally regulates energy balance and food intake through select hypothalamic structures [2–4, 6–8, 10, 12]. Disruptions in this signaling can occur with genetic disorders or acquired hypothalamic damage, both of which can frequently lead to pathologic hunger (i.e., hyperphagia), resulting in subsequent obesity and a pervasive, negative impact on quality of life [2, 3, 6–8, 10, 12, 13]. As compared with other overeating behaviors or “food noise,” patients with hyperphagia have a marked preoccupation with food and abnormal food-seeking behaviors that result from a shortened duration or failure of satiety following food intake [2, 4, 13–16]. While these and other characteristics can distinguish hyperphagia from other overeating behaviors, hyperphagia remains underrecognized and as a result is underdiagnosed [3, 13, 15]. In 2012, the 2nd International Conference on Hyperphagia was convened to focus on ongoing and future research opportunities regarding hyperphagia, primarily in the context of Prader-Willi syndrome (PWS) [4]. Continued research investigating the MC4R pathway has increased the understanding of the biology of hyperphagia. These advancements, combined with an increased availability of genetic testing, draw attention to the need for a standardized definition that can distinguish hyperphagia from other overeating disorders to support improved diagnosis and management of affected patients [4, 15]. In response to this need, an international group of physicians and scientists with expertise in hyperphagia convened in October 2023 during ObesityWeek, the annual meeting of The Obesity Society. The group discussed the need for a clear definition, a practical and accurate approach for screening and identification of affected patients, and management strategies for hyperphagia in patients with MC4R pathway–associated diseases [15]. This work serves as a summary of the proceedings from this discussion and provides potential approaches to improve the recognition and management of hyperphagia in patients with these underlying diseases.

### The Biology of Hyperphagia

**Fig. 1:**
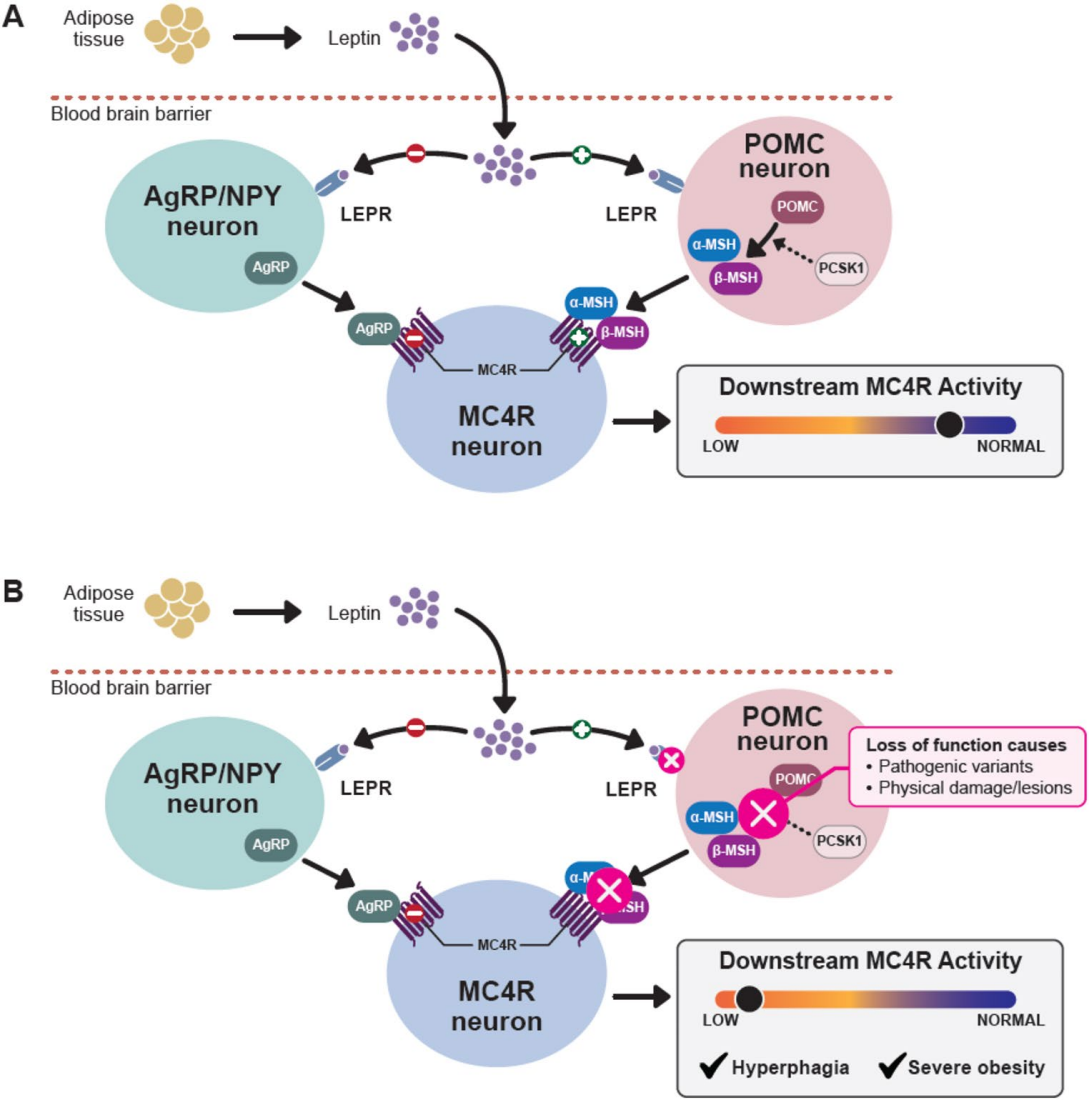
The hypothalamic melanocortin-4 receptor (MC4R) pathway. (**A**) MC4R signaling under physiological conditions. (**B**) Disruption of MC4R signaling

Research investigating hunger and energy expenditure has identified the MC4R pathway within the hypothalamus as a key regulator of these functions. This pathway and its contribution to food intake have been extensively reviewed elsewhere (e.g., Baldini and Phelan 2019) [17]. Studies of genetic and acquired impairments of the MC4R pathway have improved the understanding of the mechanisms that underlie hyperphagia [1–4, 6, 7, 10, 17, 18]. Briefly, under physiological conditions, upstream activation of MC4R by release of α–/β–melanocyte-stimulating hormone (α-/β-MSH) from hypothalamic POMC-expressing neurons decreases food intake and increases energy expenditure; these effects are balanced through inhibition of MC4R by agouti-related peptide (AgRP), an inverse agonist, released by hypothalamic AgRP-expressing neurons [4, 7, 10, 12, 17, 19, 20] (Fig. 1A). Deficiencies in upstream generation of α-/β-MSH can occur through a variety of mechanisms, including variants in genes involved in the MC4R pathway (Fig. 1B) and those that contribute to the formation of the BBSome, a collective group of proteins that guide cilia formation and regulate LEPR trafficking [20–22]. Additionally, stimulation of serotonin receptor 2 C expression in POMC neurons can further regulate MC4R signaling by enhancing POMC-mediated release of α-MSH and suppressing inhibitory AgRP activity on MC4R receptors [23, 24]. Thus, alterations in expression or splicing of serotonin receptor 2 C, potentially resulting from loss of paternal expression of *SNORD116* (as in PWS) and *BBS1* (as in BBS) could further dysregulate MC4R signaling [1, 18, 24–26]. Impairments in these upstream components of the MC4R pathway ultimately result in hyperleptinemia, in the context of leptin resistance resulting from LEPR deficiencies, and hyperinsulinemia, facilitating the development of hyperphagia and increased energy intake [9, 11, 12, 27, 28].

Variations in the underlying pathophysiology and resulting impairment of MC4R pathway signaling influence the severity of symptoms and behaviors of hyperphagia [6, 10, 12, 29]. While symptoms of hyperphagia have been observed to cluster across underlying pathologies, genetic variants that may result in hyperphagia can be associated with differing severity of functional impacts to MC4R signaling [6, 10, 30]. Additionally, these presentations can be confounded by a level of neurocognitive impairment within individuals with a specific underlying disease [4, 14, 31]. Thus, the presentation of hyperphagia does not always strictly follow such pathology-specific distinctions and there can be significant overlap of the symptoms of hyperphagia among the diseases that underlie this condition [15].

### Defining Hyperphagia

A variety of obesity-related diseases can present with hyperphagia, each with a cluster of common symptoms related to the underlying pathology (Table 1). Given the wide range in symptoms and severity, there is a need to establish a clear lexicon that includes both a general definition of hyperphagia along with separate expansions to differentiate underlying pathologies [15, 31]. Such a definition must take into consideration the age of the patient and the impact of cognitive status [4, 5, 15]. Although symptoms overlap between diseases, hyperphagia with PWS is considered the most severe presentation and is characterized by a relentless, overwhelming preoccupation with food (i.e., food noise), extreme hunger, and obsessive or unusual behaviors regarding food (e.g., waking at night to eat, eating nonfood items) [1, 4, 15, 31–34]. Patients with PWS can also experience extreme anxiety around food and exhibit tantrums, outbursts (especially during attempts at food restriction) [1, 4, 15, 33], and compulsive behaviors [31, 32]. These symptoms likely result from the underlying etiology of PWS, namely deletion of the paternally inherited chromosome 15q11-q13 region (and rarely microdeletions including the *SNORD116* gene cluster), or duplication of maternally inherited chromosome 15, which affects the expression of several maternally imprinted genes [1, 4, 18, 31, 32]. Although the presentation of hyperphagia in this population is considered severe, these symptoms should still be taken into consideration when defining hyperphagia while acknowledging they may not represent hyperphagia outside of PWS [15].

**Table 1:** The Potential Mechanism and Related Symptoms of Hyperphagia Across Underlying Pathologies

Pathology	Potential mechanism	Symptoms
PWS [1, 4, 15, 18, 24, 26, 31–33]	• Deletion of paternally inherited chromosomal region 15q11-13 or duplication of maternal chromosome 15 impacts expression of several maternally imprinted genes involved in direct or indirect regulation of hunger • Potential dysregulation of MC4R signaling resulting from serotonin receptor dysregulation resulting from absence of *SNORD116* gene cluster	• Severe preoccupation with food • Lack of satiety or short duration to satiety • Abnormal food-seeking behaviors (hoarding, hiding food, sneaking food) • Waking at night to eat • Difficulty with work/school • Tantrums, irritability, outbursts over attempts at food restriction • Eating nonfood items • Death from choking, gastric perforation with excessive or rapid eating
POMC, LEP, and LEPR deficiencies [7, 10, 13, 35]	• Decreased expression of α- and β-MSH resulting from pathogenic variants in the MC4R pathway	• Preoccupation with food • Lack of satiety or short duration of satiety • Sneaking food • Lack of control with food consumption • Difficulty with concentration and focus at work/school due to preoccupation with food or hunger
BBS [2, 3, 20–22]	• Pathologic variants in *BBS* genes impact formation of the BBSome and impair trafficking of LEPR to cell membrane, decreasing MC4R signaling	• Abnormal food-seeking behaviors and preoccupation with food • Lack of satiety and difficulty with meal termination resulting from decreased sensitivity to stomach distension • Difficulty with concentration and focus at work/school due to a preoccupation with food • Emotional dysregulation when denied access to food (i.e., tantrums, irritability)
Hypothalamic obesity [9, 11, 28, 36–42]	• Dysregulated circulating α-MSH due to damage of central hypothalamic centers that regulate its production	• Preoccupation with food • Disturbed satiety resulting in increased food intake and meal/snack frequency • Total caloric intake may be increased or normal because of decreased energy expenditure (relative hyperphagia)

α-MSH, α-melanocyte stimulating hormone; BBS, Bardet Biedl syndrome; LEPR, leptin receptor; MC4R, melanocortin 4 receptor; POMC, proopiomelanocortin; PWS, Prader-Willi syndrome

More generally, hyperphagia involves distinct characteristics that include prolonged time to satiation when eating, shortened duration of satiety following a meal, prolonged feelings of hunger, and a persistent preoccupation with food [4, 43]. Hunger and preoccupation with food caused by hyperphagia can lead to abnormal food-seeking behaviors that include searching for, negotiating or arguing for more food directly after a large meal; rapid eating; hiding or hoarding food; being distressed with denial or unavailability of food; overeating, sneaking, or stealing food; eating discarded or rotten food; and, in extreme cases, eating non-food items (e.g., paper, carpet) [1–4, 14, 15, 31, 43]. Additionally, among the most common features of hyperphagia is waking at night for food [3, 4, 14, 15, 31]. Such symptoms of hyperphagia can also lead to significant, constant, and multifaceted impacts on quality of life [2–4, 13, 15]. In studies of patients with monogenic or syndromic obesity, patients and caregivers report that hyperphagia negatively impacts sleep, mood, productivity at work or school, leisure and recreational activities, social interactions, and relationships with family and friends [2, 13, 34].

Taking into consideration the commonly observed symptoms of hyperphagia across underlying conditions, in addition to the related challenges around diagnosis and the separation of this condition from other overeating behaviors (as outlined below), a standard definition for hyperphagia is paramount [15]. Given that hyperphagia extends from aberrant physiology [6–8, 10, 12, 43] and that individuals present with distinct behaviors related to food [2, 4, 13, 14], the proposed standard definition by the authors is that hyperphagia is an insatiable, pathologic desire to consume food that is accompanied by abnormal food-seeking behaviors [15].

### Current Challenges Surrounding the Recognition of Hyperphagia

#### Overlapping Symptoms Between Hyperphagia and Overeating Behaviors

Patients with hyperphagia resulting from dysregulation of MC4R signaling are an underserved population, as the presentation of hyperphagia symptoms in a clinical setting is underrecognized. The hyperphagia can be overlooked given the overlap in symptoms with those of other overeating behaviors [4]. There is a range of severity of different overeating behaviors, and their detailed descriptions have been reviewed previously [4, 44]. Briefly, these include occasional overeating (as occurs with celebratory meals and special occasions); hedonic and emotional overeating in response to stress or boredom, or for pleasure; binge eating as marked by periodic episodes of loss of control with excess food consumption in the presence of satiety; and hyperphagia observed as an insatiable desire to consume food that is accompanied by impaired satiety and abnormal food-seeking behaviors. A diagnosis of hyperphagia can be obscured by the shared features that frequently accompany more commonly observed disorders of overeating, such as eating beyond satiety, large volume of food consumption, and loss of control of eating. However, except for the case of food insecurity [45], overeating behaviors generally do not involve the pathologic and persistent hunger that is characteristic of hyperphagia [46].

Additional distinctions of hyperphagia versus other overeating behaviors may be described as the “wanting” versus “liking” of food [8, 47]. The wanting of food is considered to be generated from a physiological salience that creates behavioral urges to seek and consume food and is neurobiologically distinct from liking of food, which is subjective in nature and stems from hedonic reward processes [8]. The neural components (mesolimbic circuitry) that give rise to wanting of food are larger than that of liking and, similarly to hyperphagia, can result in intense hunger without a respective increase in the liking of food. Additionally, the aberration of such systems can enhance food cue sensitivity or food noise, again increasing wanting without an impact on liking of food [8, 14]. Interestingly, the MC4R pathway involves bidirectional connections to both reward (liking) and homeostatic (wanting) systems, and the “liking” of food is also under homeostatic regulation (e.g. changes with nutritional state) highlighting the interconnected nature of these concepts and potentially providing an explanation of overlap between various overeating behaviors and symptoms of hyperphagia with respective dysregulation [8, 48].

### Complications in Identifying Hyperphagia

In certain situations, hyperphagia may not be considered abnormal depending on socioeconomic factors including cultural differences, health literacy, and economic standing [15, 49, 50]. Additionally, parents of a single child or new parents may not recognize abnormal behaviors in their children because they do not have a basis for comparison [15]. Each of these factors can prevent patients or caregivers from identifying or accurately reporting concerns related to eating behaviors.

Hyperphagia may also go unnoticed in cases where parents or caregivers have implemented compensatory lifestyle and dietary restrictions. Caregivers may implement methods for limiting food intake in daily living to cope with ongoing behaviors such as restricting food quantity, adhering to a low calorie diet, setting strict meal times, and locking away food [2, 13, 15, 33, 51]. Similarly, patients with cognitive impairment living in residential homes and treatment facilities are often under strict regulation involving restricted food access in addition to structured routines [4, 33]. Such modifications can result in the inability to exhibit overeating behaviors and sometimes lessen the severity of the emotional impacts of hyperphagia (i.e., aid in reducing anxiety) [4].

Adult patients without cognitive impairment who have never experienced the absence of hyperphagia may have similarly developed methods to prevent themselves from engaging in overeating behaviors and may not recognize their condition as abnormal [2, 13, 15]. Examples of this behavior have been reported as locking car keys away at night to prevent going out to get food, locking food in cupboards, placing food in alternative areas so that it could not be accessed, not purchasing unhealthy foods, and setting strict rules to limit access to unhealthy foods [15]. Patients with higher neurocognition and good health literacy may also adopt healthy copying mechanisms to compensate for the absence of normal meal termination stimuli. Patients have reported implementing strict weighing/measuring of food, calorie tracking, and reliance on social cues (i.e., watching how much others eat or when they stop eating) to determine appropriate food intake [13, 15].

Challenges are also observed within the clinic because physicians often may not be aware of hyperphagia. Within the pediatric clinical setting, overeating may be mistaken by parents, caregivers, and physicians as an indication of a healthy appetite that is appropriate for a growing child [15, 49]. However, physicians who do recognize abnormal eating or food-seeking behaviors may face challenges with performing a comprehensive assessment during standard clinic visits, given the complex nature of a hyperphagia diagnosis [15]. These challenges can include lack of time, necessary equipment, and standardized comprehensive instruments to assess hyperphagia. While self-reports of hyperphagia may provide some insight for diagnosis, these measures may not be accurate in some populations, and physicians may generally not have been trained on the appropriate questions to ask [4, 31]. Certain underlying conditions, such as BBS and PWS, are frequently accompanied by cognitive impairment, often preventing the ability for self-report [1, 3, 31–33]. Additionally, it is important to note that many patients with hyperphagia do not have a comparison to normal hunger and have not experienced the absence of hyperphagia, leading to a self-report that may underestimate the severity of the condition [2, 13, 15]. Pediatric patients or patients with cognitive impairment may not be able to articulate their experience [31]. Furthermore, adults with hyperphagia may be unwilling to share accurate descriptions of overeating symptoms because of embarrassment, guilt, obesity stigma, or feelings of blame from healthcare professionals [15]. Regardless of the challenges with these methods, self-reports by patients and caregivers can provide an understanding of the associated challenges and impacts of hyperphagia [31, 33, 52].

### Suggested Strategies for Improving Diagnosis

#### Development of Educational Resources

Education is a key element that can improve the recognition and diagnosis of hyperphagia, as knowledge of underlying pathology by both physicians and patients/caregivers can inform appropriate questions to begin a conversation around this condition [15]. Educational resources may have a multifaceted impact across individuals dealing with hyperphagia [15]. This knowledge can help clarify that hyperphagia is an aberration of normal compensatory behaviors brought on by perturbations in physiological homeostasis (e.g., prolonged food deprivation or hypoglycemia) [4, 45]. Understanding underlying pathophysiology can also improve sensitivity or unconscious biases and stigma around eating behaviors and obesity, in turn enabling patients to overcome any lack of willingness to share their concerns or discuss their experiences with overeating. Physicians and parents or caregivers can be made aware of the signs or symptoms that warrant attention and may indicate the need for further examination, although education of parents should generally be restricted to cases in which the patient is likely to manifest hyperphagia (i.e., in those with confirmed genetic variants, early-onset obesity, or a family history of severe obesity).

Educational resources should provide a detailed but accessible description of the etiology as well as associated symptoms of both hyperphagia and potential underlying diseases. It should be made clear that hyperphagia and its related behaviors cannot be willfully dismissed or overcome given that they are driven by an underlying pathology (i.e., such behaviors do not result from being “weak willed” or having a lack of self-control) [1, 2, 4, 6, 7, 10, 15, 29]. While these topics should apply to healthcare professionals and patients or parents and caregivers, separate educational materials should be created for each of these groups. Regarding patients or parents and caregivers, education should inform individuals of abnormal food-seeking behaviors and strategies to help patients manage symptoms, such as food restriction, food security and safety, and increased physical activity [4, 15]. Regarding physicians, educational resources should additionally include approaches for screening for hyperphagia to be cognizant of the many factors that may obscure responses.

#### Efficient Clinical Screening

Although instruments for assessing hyperphagia, such as the Dykens Hyperphagia Questionnaire, have reported higher hyperphagia scores in those with obesity associated with *LEPR* and *MC4R* variants compared with those with syndromic obesity [30], further investigations are necessary to verify to what extent current questionnaires will be suitable for patients with hyperphagia resulting from rare MC4R pathway–associated diseases [5, 13, 53]. Additionally, while several eating behavior questionnaires have been used to assess hyperphagia, these are often not easily implemented in routine clinical visits [4, 13, 15, 31, 54, 55]. Thus, a set of simple questions may be best suited for identifying patients with hyperphagia in a general clinic setting [15]. Such questions should have a narrow focus on the most distinct characteristics and concepts of hyperphagia and assess the objective features of the related symptoms (Table 2).

**Table 2:** Distinct Characteristics of Hyperphagia and Related Features

Characteristic	Features
Prolonged time to satiation	Size of a meal, calories needed to reach fullness, meal termination
Shortened duration of satiety	Abnormal postprandial satiety, short intermeal interval
Prolonged feelings of hunger	Desire to eat, persistent hunger, eating behaviors and emotions elicited by food availability, abnormal food-seeking behaviors

These characteristics and related features can be driven by the difficulty to feel full [15, 56]. A computational model has shown that fullness can predict intermeal intervals and meal termination and when deficits within the MC4R pathway (i.e., alterations to AgRP neurons or leptin) were simulated, fullness, intermeal interval, and meal termination all varied predictively [56]. Such findings highlight the relationship of the MC4R pathway in regulating these components of feeding behavior and hunger and offer credibility for the use of these features to assess for abnormalities, such as hyperphagia. Simple yes/no or short-answer questions can be tailored around these features of hunger to allow patients to correctly interpret what is being asked and allow for patients to accurately relay their experiences [15]. However, as patients with hyperphagia are often not familiar with normal hunger or fullness [2, 13], these concepts should be defined for patients (i.e., relief from anxiety, change in physical sensation, dissipation of wanting food) before screening [15]. Questions should include feelings around food, the duration and change in feeling with a meal, and the impact of hunger on daily living (Table 3) and should be asked in the context of patient history and current feelings (i.e., “have you ever” and “do you ever”).

**Table 3:** A Proposed Set of Questions to Screen for Hyperphagia [15]^,a^

**Questions of satiety**
• Do you ever feel full and how long does this feeling last? − How long after a meal do you feel hungry again? − Do you eat past feeling full? • What causes you to stop eating? • How long do you usually go between meals? • Can you skip meals? • How often do you eat meals/snacks?
**Preoccupation with food**
• Are you anxious if food is restricted or withheld from you or if you cannot find or access food? • Is food locked away from you by your spouse, parents, or roommates? • Do you miss out on certain activities or hobbies you otherwise like because you are thinking of food or eating?
**Abnormal food behaviors**
• Have you ever eaten food out of the garbage or rotten food? • Have you ever eaten things that are not usually considered food, like paper, crayons, or carpet? • Have you hidden, hoarded, or eaten food in secret? • Do you wake up at night to eat food? • Have you ever stolen food or stolen money to buy food? • How often is disturbed eating behavior present: always, sometimes, exceptionally, never?

^a^Questions should be updated to accommodate “your child” for the case of parent’s and/or caregiver’s report

In addition to inquiring about physical sensations and food intake, questions should also gather information about the methods put into place to overcome symptoms of hyperphagia (e.g., “Do you take actions to prevent yourself from consuming food?”) [15]. Such questions can provide a more sensitive measure of hyperphagia and are objective in nature, which can help identify patients who have developed strict coping mechanisms who may otherwise pass unnoticed. Parents or caregivers should serve as proxy in cases of patients with cognitive impairment or those who are too young to reliably self-report though observational responses [3, 5, 15, 55]. Overall, the recognition of hyperphagia requires a multifaceted approach that includes education, carefully chosen directed questions, and quantitative assessments and should be followed up with a diagnosis of underlying pathology to inform targeted impactful treatment strategies [3, 4, 54].

### Assessments of Hyperphagia

#### Questionnaires

Following screening, patients should be referred for genetic testing to determine or confirm underlying etiology and should undergo in-depth assessments that can include expanded age-appropriate questionnaires tailored to varied presentations resulting from distinct diseases. Comprehensive assessments of hyperphagia, such as validated questionnaires and other quantitative measurements, should also be performed because they can provide a means of determining severity and also aid in the diagnosis of underlying conditions in cases where genetic testing is unable to confirm a diagnosis [4, 15, 54]. The variety of questionnaires currently used by physicians to assess eating behaviors have been previously reviewed [4] and include the Dykens Hyperphagia Questionnaire [31] (developed for PWS; assesses the frequency and severity of behaviors); the Hyperphagia Questionnaire for Clinical Trials (developed for clinical trials of PWS) [51], the Three-Factor Eating Questionnaire [54]; Dutch Eating Behavior Questionnaire [57]; the Childrens Eating Behavior Questionnaire [55]; Food-Related Problems Questionnaire [58]; Power of Food Scale [59]; Yale Food Addiction Scale [60]; and the Binge Eating Scale [61]. However, although certain characteristics of hyperphagia may be assessed by current questionnaires [5, 15, 62], several have not been designed to directly assess hyperphagia [30, 55, 61], and existing questionnaires have not been validated for assessment of MC4R pathway–associated diseases [5, 15]. Therefore, a standard questionnaire for examining hyperphagia has not been established, and physicians vary in which tools they use clinically for evaluating this condition.

The use of disparate questionnaires in different clinics and studies can confound comparisons, and several important considerations should be acknowledged when reviewing questionnaire responses. Among current questionnaires, repetitive recording of food intake or hyperphagic behaviors over long periods can be difficult for patients, the overfamiliarity that may occur with daily questionnaires can result in rote responses [15]. Responses to questionnaires may also vary based on whether they are reported by caregivers or are self-reports from patients [63, 64]. Caregiver reports are often necessary for patients with cognitive impairment or in those who are too young to provide a reliable response [15, 63]. However, caregivers may inflate the severity of hyperphagia given that symptoms may seem more extreme than what is perceived by a patient who has never experienced the absence of hyperphagia [15]. Studies have reported a lack of overlap between patients and respective caregivers with questionnaires adapted for both patient and caregiver/parent versions, likely resulting from the inability of a caregiver to experience the internal state of a patient [3, 15, 31, 63, 64]. In contrast, self-reports of hyperphagia may underrepresent the severity of symptoms because hyperphagia may be the only state some patients have experienced and they have adapted to living with the condition or may not recognize behaviors done unintentionally [2, 31, 63, 64]. Self-reports from patients who developed hyperphagia after injury to hypothalamic regions, such as in acquired hypothalamic obesity, who can recall normal hunger may provide a unique insight into the lived experience of this condition and its comparison to normal hunger [15]. Finally, patients may respond differently when treated with medications that increase satiety, such as antiobesity medications (AOMs) and neuroleptics [4, 15, 33], and previous diet counselling or restrictions can additionally limit the natural history of hyperphagia and modify food-seeking behavior [63]. Despite these challenges and considerations, self-reported questionnaires may create an environment in which patients are more comfortable describing their behaviors and challenges without having to directly admit or discuss them with a physician, and efficacy of the questionnaires is often increased if provided before a clinical visit [15].

#### Quantitative Measurements of Hyperphagia

Beyond subjective qualitative questionnaires, several quantitative measurements have also been used to assess hyperphagia. Ad libitum meals, or observation of food intake can reveal emotional distress around food presentation, how patients eat, and the amount of food consumed [13, 15, 30, 31, 55]. However, the lack of standard methodology that results in variations of testing conditions (i.e., fasted or fed state, the palatability of the food presented, and whether or not the same food is presented for multiple meals) can affect the results of these tests [15]. Further, these approaches may not be ideal to implement in a clinical practice setting because they can be onerous and intensive to perform, and it has been stated that providing ad libitum access to food is unethical in patients with severe hyperphagia [4, 15, 31].

The use of functional magnetic resonance imaging (fMRI) may be another useful objective tool for assessing hyperphagia. Regional brain activity in response to food cues (e.g., pictures) has been observed with fMRI [16, 65–67]. This method can assess changes in response to treatment and can bypass the inherent noise of subjective questionnaires. Brain regions that have exhibited changes in the context of food included the ventral and dorsal striatum, insula, amygdala and orbitofrontal cortex, as shown in children with common obesity [8, 42, 68]. In patients with PWS, alterations of functional connectivity between areas of reward processing, motivation, and the motor sensory network have been observed, as has aberrant neural responses to visual food stimuli before and after a meal in limbic and paralimbic regions that drive eating behavior [69, 70]. Additionally, patients with leptin deficiency have exhibited differential bilateral activity within the substantia nigra/ventral tegmental area, amygdala, and orbitofrontal cortex [68, 71], along with altered activation of the ventral striatum [62]. Findings with fMRI also extend to patients with hypothalamic obesity, in which patients with a history of craniopharyngioma treatment and obesity have exhibited altered activation in the nucleus accumbens and medal orbitofrontal cortex in response to food cues, compared with controls [67]. This method may not be readily accessible by some clinicians and may be additionally restricted if a wide bore MRI scanner is needed for a patient with increased weight. Furthermore, analysis of fMRI data of food cue reactivity typically requires averaging across multiple individuals rather than examining individual patient responses [72, 73]. Thus, while fMRI and related tools are available for research of hyperphagia, they may also be impractical for use in a routine clinical setting, and such use is not fit for diagnosing hyperphagia because of large variability in response.

#### Assessing Hyperphagia in Patients with MC4R Pathway–Associated Diseases

The challenges faced with current comprehensive assessments of hyperphagia further highlight the need for a standardized hyperphagia questionnaire tailored for MC4R pathway–associated diseases [4, 5, 15, 54]. A consensus on a hyperphagia lexicon could be readily adapted into a hyperphagia questionnaire tailored for MC4R pathway–associated diseases. A standardized questionnaire should be adaptable to expand across varying underlying pathologies and take into consideration family and medical history, including body mass index [15]. Broader subjective, impact-related questions about social interactions, productivity, and engagement in leisure or recreational activities could also aid in diagnosis. However, these questions should be included secondary to a standardized questionnaire and should not impact quantitative assessments. Once established, standardized hyperphagia questionnaires can also be readily adapted to monitor response to treatment with objective measurements of hunger control, thus informing potential management strategies and the efficacy of varied therapeutic approaches [3, 5, 13, 15, 54, 58]. The recent advancements in understanding hyperphagia have led to the development of Symptoms and Impacts of Hyperphagia Questionnaires that are each tailored for patients with hyperphagia resulting from MC4R pathway–associated diseases, such as BBS [53]. While most questionnaires focus on the addictive or compulsive nature associated with hyperphagic behaviors [58–60], these novel questionnaires were designed to record the frequency of common behaviors of hyperphagia (e.g., sneaking food, waking at night for food) and how those behaviors impact daily living [53]. Further, these questionnaires do not include the extreme symptoms of hyperphagia that are incorporated into hyperphagia questionnaires developed for PWS [53]. The Symptoms and Impacts of Hyperphagia Questionnaires have been shown to provide a good correlation with existing tools while also reporting a higher fidelity among reports from patients with BBS and their caregivers [53], and they have additionally shown improvement in hyperphagia symptoms with treatment for MC4R pathway–associated diseases [2, 13].

### Treating Hyperphagia

#### Goals of Treatment

Assessment of treatment response would also benefit from a consensus around the definition of hyperphagia and the deviation from normal hunger because a primary objective of treatment should be to address hunger [2, 15]. Management of weight gain should also be a primary concern and be prioritized over drastic weight loss, as individual patients with obesity may have a higher set point for their weight or fat mass [15, 29]. Additional goals should include controlling hyperphagic behaviors through restricting access to food and establishing and maintaining a dietary plan and healthy habits and a good physical activity regimen [4, 15]. Such goals can be achieved through a coordinated comprehensive approach to care [15, 74]. However, training of physicians on the underlying pathologies of weight and hunger may be necessary to overcome routine treatment plans for patients with obesity-related diseases as well as the pervasive prevalence of biases and stigma around the use of medications in these patient populations [15].

#### Considerations for Treating Patients with MC4R Pathway–Associated Diseases

When considering treatment approach, it is important that treatment of hyperphagia should occur early, be sustained, and target the underlying pathology. Early intervention, particularly in young children, can prevent the morbidity caused by the future development of obesity-related diseases (e.g., type 2 diabetes mellitus, cardiovascular disease, fatty liver disease) and may reduce the stigma associated with weight gain [13]. As an example of the necessity of a targeted approach, bariatric surgery in patients with MC4R pathway–associated diseases often results in transient weight loss that is frequently regained [4, 7, 12, 75]. This treatment approach does not address the underlying deficit in α-/β-MSH signaling that causes the inappropriate food intake and energy expenditure, which are effects that persist after bariatric surgery [15, 36, 38, 41]. Treatments that target underlying pathology can improve hunger while also regulating weight and metabolic parameters, thus decreasing the risk for future disease [35, 41, 76, 77]. Such effects have been observed in patients with rare MC4R pathway–associated diseases, such as LEPR deficiency, POMC deficiency, and BBS who have been treated with the MC4R agonist setmelanotide, which was additionally associated with rapid improvements in hunger. For further emphasis of addressing underlying etiology and the need for long-term treatment, data from pivotal trials of setmelanotide in these patient populations have shown that interruption of discontinuation of treatment led to a rebound weight gain [35, 76]. Although not directly assessed, increased weight gain may result in part from a recurrence of hyperphagia. Overall, a clear understanding of disease etiology supplemented with a standard treatment approach and assessment of treatment effect on hyperphagia is imperative to improve access to effective treatment strategies and better serve patients with this condition [3, 29].

#### Current Treatments for Hyperphagia

Current treatment strategies for hyperphagia are varied and include lifestyle modifications, food restriction, deep brain stimulation, bariatric surgery, AOMs (e.g., stimulants, glucagon like peptide 1 receptor [GLP1] – and glucose insulinotropic peptide [GIP]–based compounds, MC4R agonists), and other pharmacotherapies (e.g., topiramate, naltrexone, oxytocin) [2, 4, 15, 74]. Effective treatment strategies have been previously reviewed and highlight the benefits of employing combination therapy [15, 74]. As an example, a patient with hyperphagia might exhibit some benefit from dietary modifications as well as experience initial weight loss with bariatric surgery and concurrent use of multiple AOMs, which could then be continued for long-term control of hunger. Physicians should take care when prescribing concomitant treatments to ensure they adequately address hyperphagia and do not overcorrect because combination therapies are additive [3, 15, 74, 78].

#### Determining Response to Treatment

Symptom improvement should be assessed on an individual basis given variability in presentation of hyperphagia, related comorbidities, and general individual response to treatment. While decreased hunger and maintenance or reduction in weight are key indicators of treatment efficacy, a successful response extends beyond this and generally would allow patients to have a relatively normal quality of life when navigating a modern food environment [2, 13, 35, 41, 74]. This type of response may present as a decrease in food noise or the constant preoccupation with food, snacking, anxiety, and psychological impact; smaller portion sizes when eating; ability to skip meals; and increased participation in activities that are unrelated to food [2, 4, 13, 15, 16]. Additional benefits of treatment may also be considered when examining an individual response, such as an increase in cognitive capacity, which could be measured quantitatively. Some patients have observed an improved focus after experiencing a reduction in their preoccupation with food [2, 15]. Patients have additionally reported being better able to concentrate at work and school or are now able to engage in extracurricular activities [2, 13]. Similarly, caregivers and the families of patients report reduced stress and improved familial relationships [2, 33]. As an example, while patients with PWS may report persistent hyperphagia with treatment, a caregiver may report absence of physical aggressiveness with food withholding, decreasing the morbidity impact of hyperphagia on caregivers [33]. Of significant importance, assessments of treatment efficacy may be most informative in the context of withdrawal from effective treatment because patients may be more able to accurately describe treatment benefit once they have experienced the absence of hyperphagia, particularly as related to a change in hunger [2, 13, 15, 16].

## Concluding Remarks and Future Directions

Recent investigations into MC4R pathway–associated diseases have improved the understanding of hyperphagia etiology; however, hyperphagia remains underrecognized and underdiagnosed [2–4, 6, 7, 9–11]. Increasing the awareness of hyperphagia and its underlying etiology can improve identification of this condition and aid in overcoming the stigma associated with overeating behaviors and obesity [15]. In order to better serve patients with improved diagnosis and management of hyperphagia, a standardized definition that provides a distinction from other overeating disorders is needed [4, 15]. Here we have proposed a general definition for hyperphagia as a pathologic insatiable desire to consume food, accompanied by abnormal food-seeking behaviors [15]. Combined with a consensus of the behaviors and symptoms that constitute hyperphagia, establishing efficient screening tools that can be adaptably expanded for the variety of underlying pathologies can help guide targeted treatment strategies for effective management of hunger and obesity [4, 5, 15]. Continued efforts toward increased education and the development of standardized definitions, an adaptable clinical screening questionnaire, and comprehensive assessments of hyperphagia hold promise to improve the management of hyperphagia and the quality of life for patients with MC4R pathway–associated diseases who experience this condition.

## Data Availability

No datasets were generated or analysed during the current study.
