# Correction to: Defining Hyperphagia for Improved Diagnosis and Management of MC4R Pathway–Associated Disease: a Roundtable Summary

**DOI:** 10.1007/s13679-025-00616-0

**Published:** 2025-03-11

**Authors:** Steven B. Heymsfield, Karine Clément, Beatrice Dubern, Anthony P. Goldstone, Andrea M. Haqq, Peter Kühnen, Jesse Richards, Christian L. Roth, Erica L. T. van den Akker, Martin Wabitsch, Jack A. Yanovski

**Affiliations:** 1 Pennington Biomedical Research Center, Louisiana State University System, Baton Rouge, Louisiana USA; 2 Assistance Publique-Hôpitaux de Paris, Nutrition Department, Pitié-Salpêtrière Hospital, Paris, France; 3 Sorbonne UniversitéInserm, Nutrition and Obesities; Systemic Approaches, NutriOmique Research Group, Paris, France; 4 Sorbonne Université, Trousseau Hôpital, Assistance Publique-Hôpitaux de Paris, Paris, France; 5 Psychoneuroendocrinology Research Group, Division of Psychiatry, Department of Brain Sciences, Faculty of Medicine, Imperial College London, London, UK; 6 Imperial Centre for Endocrinology, Imperial College Healthcare NHS Trust, Hammersmith Hospital, London, UK; 7 International Network for Research, Management & Education on Adults with Prader-Willi Syndrome (INfoRMEd-PWS), London, UK; 8 Department of Pediatrics, Division of Pediatric Endocrinology, University of Alberta, Edmonton, AB Canada; 9 Department of Pediatric Endocrinology and Diabetology, Charité - Universitätsmedizin Berlin, Corporate Member of Freie Universität Berlin und Humboldt-Universität zu Berlin, Berlin, Germany; 10 German Center for Child and Adolescent Health (DZKJ), Partner Site, Berlin, Germany; 11 Department of Internal Medicine, University of Oklahoma at Tulsa, Tulsa, Oklahoma USA; 12 Seattle Children’S Research Institute, Seattle, WA USA; 13 Division of Endocrinology, Department of Pediatrics, University of Washington, Seattle, WA USA; 14 Erasmus University Medical Center, Rotterdam, The Netherlands; 15 Division of Pediatric Endocrinology and Diabetes, Department of Pediatrics and Adolescent Medicine, University of Ulm, Ulm, Germany; 16 Section on Growth and Obesity, Division of Intramural Research, Eunice Kennedy Shriver National Institute of Child Health and Human Development, National Institutes of Health, Bethesda, Maryland USA

**Correction to: Curr Obes Rep (2025) 14:13**

10.1007/s13679-024-00601-z

**STATEMENTS AND DECLARATIONS**

**Competing interests**

All roundtable participants received honorarium and travel reimbursements from Rhythm Pharmaceuticals, Inc. Authors were not paid for their participation in this manuscript.

**SBH** is a medical advisory board member for Tanita Corporation, Amgen, Novo Nordisk, Versanis Bio, and Medifast and has served as an Amazon Scholar

**KC** is a primary investigator for Rhythm Pharmaceuticals, Inc. and her research group has received research support from Rhythm Pharmaceuticals, Inc.

**BD** is a consultant for Novo Nordisk and primary investigator for Rhythm Pharmaceuticals, Inc.

**APG** has been a consultant for Evidera, Helsinn Healthcare, Idera Pharmaceuticals, Rhythm Pharmaceuticals, Inc., Soleno Therapeutics, Tonix Pharmaceuticals, and Veda Ventures; has been an advisory board member for Millendo Therapeutics and Radius Health; has been a member of the Data Safety Monitoring Committee for Novo Nordisk; has received speaker honoraria from Novo Nordisk and Rhythm Pharmaceuticals, Inc.; and has been a Principal Investigator for clinical trials sponsored by Millendo Therapeutics, Rhythm Pharmaceuticals, Inc., and Soleno Therapeutics.

**AMH** has received grants from the Weston Family Microbiome Initiative and Canadian Institutes of Health Research and is an advisory board member for Rhythm Pharmaceuticals, Inc., the 2023 Novo Nordisk Pediatric Expert Obesity National, and Foundation for Prader-Willi Research USA. She is PI for clinical trials with Rhythm Pharmaceuticals, Inc., Acadia Pharmaceuticals, NovoNordisk, and Eli Lilly.

**PK** has participated on a safety board for and their institution has received funding for clinical trial research from Rhythm Pharmaceuticals, Inc.

**JR** is on speaker bureaus for Eli Lilly, Novo Nordisk, and Rhythm Pharmaceuticals, Inc. and is an independent consultant for Palatin Technologies.

**CLR**’s institution has received research support from Rhythm Pharmaceuticals, Inc.

**EvdA**’s institution has received funding for clinical trial research from Rhythm Pharmaceuticals, Inc.

**MW** has received consulting fees and payment for educational events/lectures from Rhythm Pharmaceuticals, Inc. and their institution has received funding for clinical trial research from Rhythm Pharmaceuticals, Inc.

**JAY** reports grant support from Soleno Therapeutics and Rhythm Pharmaceuticals, Inc. for obesity-related projects as well as material support for research from Hikma Pharmaceuticals plc and Versanis Bio.

Regarding the article, Defining Hyperphagia for Improved Diagnosis and Management of MC4R Pathway–Associated Disease: A Roundtable Summary, the authors were informed after publication that two mentions of the gene SNORD116 would be more accurate if updated to SNORD115 (1). While the current reference does support SNORD116 having a role in food intake/body weight, the serotonin receptor regulation is reported specifically with SNORD115.

(1) Qi Y, Purtell L, Fu M, Lee NJ, Aepler J, Zhang L, et al. Snord116 is critical in the regulation of food intake and body weight. Sci Rep. 2016;6:18614. 10.1038/srep18614

